# A Severe Form of Atypical Hemolytic Uremic Syndrome in a Two-Year-Old Girl: A Case Report

**DOI:** 10.7759/cureus.60502

**Published:** 2024-05-17

**Authors:** Ashwin Meshram, Ritu Rajan, Ishani Arora, Shruti Dange, Abhiram Chandran

**Affiliations:** 1 Pediatrics, Datta Meghe Medical College, Datta Meghe Institute of Higher Education and Research (Deemed to Be University), Nagpur, IND; 2 Nephrology, Datta Meghe Medical College, Datta Meghe Institute of Higher Education and Research (Deemed to Be University), Nagpur, IND

**Keywords:** plasmapheresis, atypical hus, microangiopathy, kidney, case report

## Abstract

Hemolytic uremic syndrome (HUS) is a prevalent cause of severe acute kidney injury in children, often leading to chronic renal damage. It is characterized by thrombotic microangiopathy (TMA), which represents a triad of microangiopathic hemolytic anemia, thrombocytopenia, and renal impairment. The choice of treatment and management strategies depends primarily on the underlying etiology. We present the case of a two-year-old girl diagnosed with rapidly progressive glomerulonephritis accompanied by hypertension necessitating renal replacement therapy. Initial laboratory findings indicated positive antinuclear antibodies, prompting immunosuppression and renal biopsy, revealing TMA with minimal chronicity changes. The treatment involved plasmapheresis and a single dose of injection rituximab, resulting in clinical recovery with an improved glomerular filtration rate. Since the anti-complement factor H antibody result was negative, the genetic etiology of atypical HUS was considered. The patient was discharged with favorable outcomes, including normal urine output and the absence of edema. This case concludes that young children with atypical HUS may present with a severe clinical course necessitating early intervention. The lack of genetic analysis facilities in severe cases should not hinder the timely initiation of plasmapheresis to prevent further injury and progression to chronic kidney disease.

## Introduction

Hemolytic uremic syndrome (HUS) stands as a significant cause of severe acute kidney injury (AKI) in childhood, with approximately one-third of patients progressing to chronic kidney disease [1]. HUS is characterized by microangiopathic hemolytic anemia, defined by anemia (hemoglobin <10 g/dL, hematocrit <30%), fragmented red cells in peripheral smear (schistocytes ≥2%), elevated lactate dehydrogenase (LDH) (>450 IU/L), or undetectable haptoglobin, thrombocytopenia (platelet count <150,000/mm^3^), and AKI, defined as a 50% increase in serum creatinine from baseline, are essential diagnostic criteria [2].

The classification of HUS is based on the etiology and pathophysiology of thrombotic microangiopathy (TMA), encompassing various forms such as Shiga toxin-associated HUS, pneumococcal HUS, infection-associated HUS, secondary HUS, defective cobalamin metabolism, and atypical HUS [3]. The term "atypical HUS" is reserved for cases devoid of co-existing diseases [3].

In the Indian population, atypical HUS associated with anti-complement factor H (anti-CFH) antibodies prevails, constituting approximately 50% of pediatric HUS cases [4]. Recent insights into the disease reveal that almost 60% of atypical HUS patients exhibit gene mutations encoding complement-regulating proteins, leading to atypical HUS presentation in children without autoimmune anti-factor H antibodies [5]. The 2015 international consensus recommends genetic screening in all atypical HUS cases (first episode or relapse) in the absence of causative diseases, Shiga toxin-producing *Escherichia coli* (STEC) infection, severe "a disintegrin and metalloproteinase with a thrombospondin type 1 motif, member 13" (ADAMTS13) deficiency, hyperhomocysteinemia, or methylmalonic aciduria [3]. The standard of care for children with atypical HUS lacking anti-CFH antibodies involves complement blockade with eculizumab, a humanized anti-complement C5 antibody [6]. However, due to cost and accessibility issues in India, plasmapheresis or plasma infusions often serve as alternatives, particularly for patients with genetic defects in the complement pathway [2]. Follow-up for at least five years is essential for monitoring hypertension, proteinuria, and glomerular filtration rate [1].

## Case presentation

In March 2023, a two-year-old girl, born of nonconsanguineous parents, was admitted with periorbital puffiness, progressing to pedal edema over six days, and decreased urine output for the same duration. Examination revealed lethargy, tachycardia (heart rate: 158 beats per minute), and stage two hypertension (blood pressure: 140/100 mmHg). Immediate laboratory investigations indicated severe AKI (serum creatinine: 13.61 mg/dL, serum urea: 320 mg/dL), hyperkalemia (serum potassium: 5.57 mEq/L), metabolic acidosis, and severe anemia (hemoglobin: 7.7 g/dL, total leukocyte count: 6100/mm^3^, platelets: 2.24 lakhs/mm^3^). Acute peritoneal dialysis (PD) with blood transfusion was initiated for stabilization.

Following initial stabilization, further evaluation was aimed at ascertaining the cause of AKI. Urinalysis revealed 3+ proteinuria and microscopic hematuria. The peripheral smear indicated anemia with occasional schistocytes but no thrombocytopenia. Normocomplementemia (serum complement C3: 87 mg/dL, serum complement C4: 0.37 g/L) was observed, and the anti-streptolysin O (ASO) titer was negative. Serum antinuclear antibody (ANA) by immunofluorescence assay returned positive (1:100) in a nuclear-speckled pattern. Cytosolic antineutrophil cytoplasmic antibody (cANCA) and perinuclear antineutrophil cytoplasmic antibody (pANCA) were negative. Ultrasound indicated a right kidney size of 5 cm and a left kidney size of 5.6 cm, with maintained bilateral corticomedullary differentiation (Table 1).

**Table 1:** Etiologic workup. ASO: antistreptolysin O, ANA: antinuclear antibody, cANCA: cytosolic antineutrophil cytoplasmic antibody, pANCA: perinuclear antineutrophil cytoplasmic antibody, anti-CFH: anti-complement factor H, mg/dL: milligrams per deciliter, g/L: grams per liter, IU/mL: international units per milliliter, AU/mL: arbitrary units per milliliter.

Test	Reference Range	Result	Interpretation
Serum C3 complement	83–192 mg/dL	87 mg/dL	Normal
Serum C4 complement	0.16–0.48 g/L	0.37 g/L	Normal
ASO titer	Less than 100 IU/mL	Less than 100 IU/mL	Negative
ANA	Less than 1:80	1:100 nuclear-speckled	Positive
cANCA and pANCA	Less than 1:20	Less than 1:20 (each)	Negative
Anti-CFH antibody	393–1069 AU/mL	25 AU/mL	Negative

Amid this initial perplexing scenario and poor improvement in urine output after 10 days of illness with ongoing acute PD, immunosuppression was initiated due to rapidly progressive glomerulonephritis. Three doses of injection methylprednisolone at 10 mg/kg body weight per day (mg/kg/day) were administered on three consecutive days, followed by oral prednisolone at 1 mg/kg/day, and a renal biopsy was performed.

**Figure 1:**
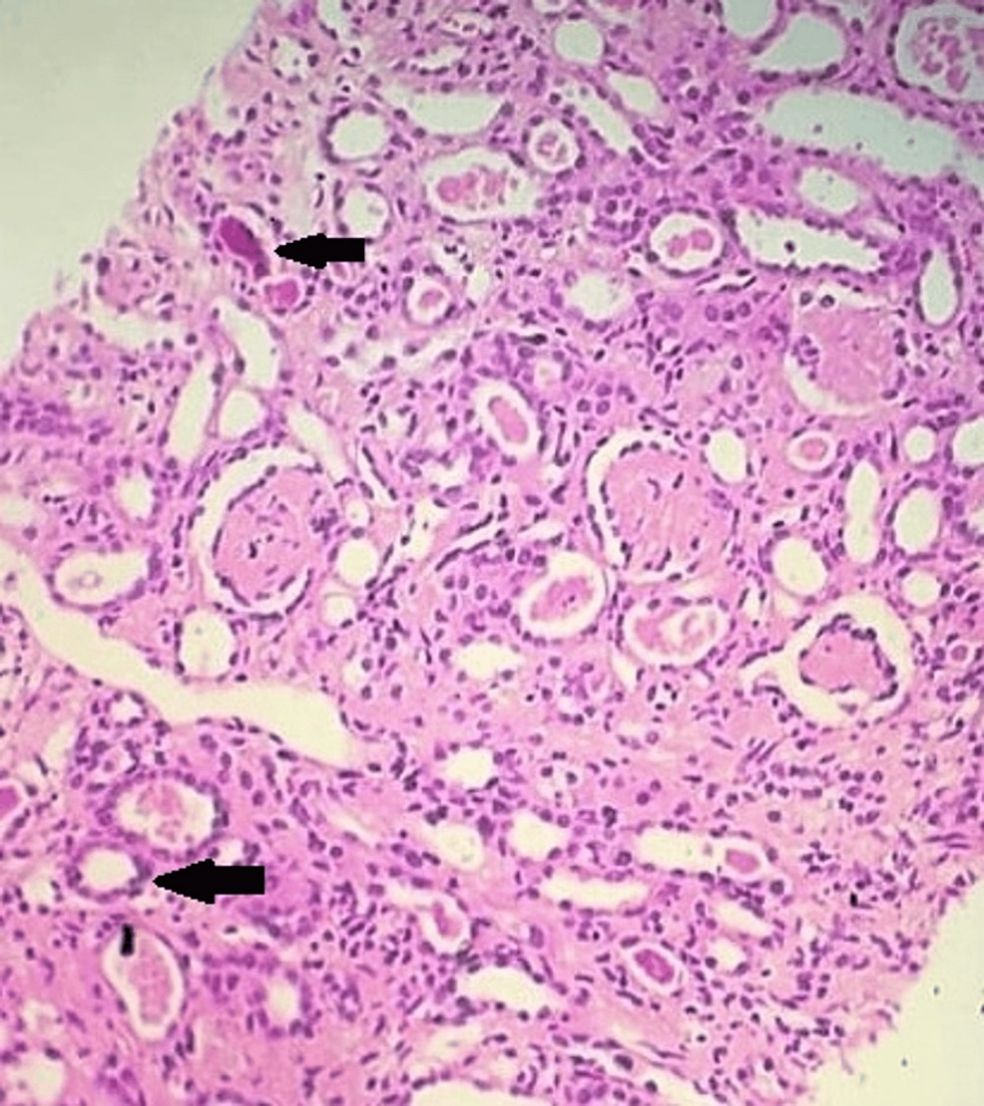
Kidney biopsy light microscopy (hematoxylin and eosin stain). The black arrows show variable ischemic changes in the cortical tissue secondary to thrombotic microangiopathy with prominent tubular injury.

Meanwhile, subsequent blood investigations suggested a decrease in hemoglobin count and the gradual appearance of thrombocytopenia. Peripheral smear indicated hemolytic anemia with schistocytes, and serum LDH levels were 1078 U/L. The renal biopsy report suggested light microscopy showing TMA with 59% (13 out of 22) glomeruli exhibiting an ischemic shrunken appearance and prominent tubular injury (Figure 1).

**Figure 2:**
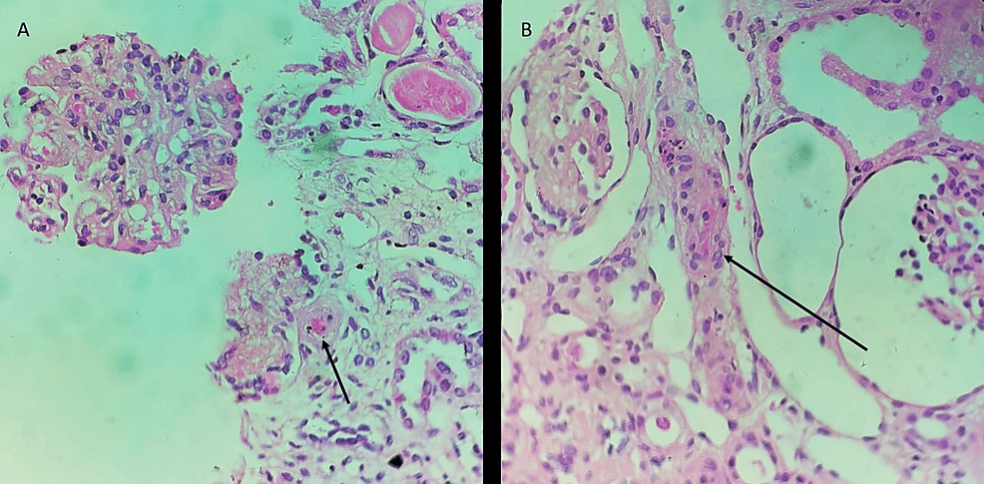
Kidney biopsy light microscopy (hematoxylin and eosin stain). A: The black arrow points toward a hilar vessel fibrin thrombus. B: The black arrow points toward the vessel changes with occlusion and fragmented red cells in the wall. Note the variable ischemic changes in the glomeruli.

A biopsy also shows glomerulus showing hilar vessel fibrin thrombus, which is known as an onion peel appearance. Moreover, vessel occlusion and fragmented red blood cells (RBCs) in the wall can be noticed in the biopsy examination (Figure 2).

Non-proliferative glomerulonephritis was observed, with no crescents or wire loop lesions. Immunofluorescence was negative. Given the biopsy report, the diagnosis of HUS was made, and plasmapheresis was planned. Serum was sent for anti-CFH antibody, and oral mycophenolate mofetil (MMF) was initiated at 750 mg/m^2^ of body surface area. Seven plasmapheresis sessions utilizing fresh frozen plasma as an exchange fluid were performed, with initially no observed clinical improvement as the child remained oliguric and dialysis-dependent. Blood reports showed no hematological remission, with hemoglobin of 6.4 g/dL, platelet count of 1.57 lakhs/mm^3^, and serum LDH of 832 U/L. Hypertension worsened, necessitating multiple oral antihypertensives, and the patient experienced hypertensive emergency and encephalopathy. Intravenous nitroglycerine (NTG) infusion was administered for 48 hours. A computed tomography (CT) scan of the head revealed features consistent with posterior reversible encephalopathy syndrome (PRES) with cerebral microthrombi.

Consequently, a single dose of injection rituximab at 375 mg/m^2^ of body surface area was administered. After two days, improvements were noted, with gradually increasing urine output, improved estimated glomerular filtration rate (eGFR), and stabilized serum creatinine levels of approximately 1.5 mg/dL. Hematological remission was also observed, with hemoglobin count improving to 10.9 g/dL and a platelet count of 3.14 lakhs/mm^3^. Anti-CFH antibody levels were 25 AU/mL, returning negative. Genetic analysis was recommended, but financial constraints prevented its execution. Currently, with controlled HUS activity post-plasmapheresis and rituximab, blood pressure is well-managed with six oral antihypertensive drugs. The child was discharged after a month-long hospital stay on oral prednisolone and oral MMF. In subsequent follow-ups, the child has exhibited positive progress with no edema or oliguria, blood reports indicating no hemolytic activity or thrombocytopenia, and serum creatinine consistently between 1.3 and 1.5 mg/dL (Table 2).

**Table 2:** Laboratory investigations during hospital stay. LDH: lactate dehydrogenase, g/dL: grams per deciliter, mm: millimeter, U/L: units per liter, mg/dL: milligrams per deciliter.

Laboratory Parameters	Reference Range	On Admission Day	Tenth Day of Admission	During Plasmapheresis	Post-plasmapheresis and Rituximab	On Discharge Day
Hemoglobin	11.5–14.5 g/dL	6.5	3.9	10.1	6.7	7.9
Platelets	1.50–4.00 lakhs/mm^3^	2.57	1.05	0.58	3.15	4.62
Serum LDH	150–450 U/L	1078	832	647	492	497
Serum urea	5.14–16.8 mg/dL	225	178	50	92	122
Serum creatinine	0.03–0.50 mg/dL	13.61	5.05	1.7	1.66	1.5

## Discussion

Atypical HUS in children can present with life-threatening complications and severe outcomes [7]. A study reported that if oliguria persisted for more than 14 days or anuria for more than seven days, the chances of renal recovery from atypical HUS were only 13%, with most children experiencing severe chronic kidney disease, end-stage kidney disease, or mortality [8]. In this case, the patient presented with severe oliguria lasting almost 20 days, with a serum creatinine at discharge of 1.7 mg/dL.

The central pathophysiology of atypical HUS involves TMA, driven by abnormalities in the alternative complement pathway, leading to endothelial cell dysfunction and microvascular thrombi formation. Platelet consumption in microthrombi causes thrombocytopenia, hindering blood flow, and the shearing of erythrocytes results in hemolytic anemia. Renal impairment in atypical HUS is secondary to similar microthrombi formation in renal vasculature [9]. Approximately 60%-70% of atypical HUS patients develop anti-CFH antibodies or carry identifiable mutations in complement genes, contributing to alternative complement pathway dysregulation [3]. These abnormalities can be identified by standardized techniques, which are expensive in India.

Eculizumab, a monoclonal antibody that works against complement C5, a key mediator in the activation of the alternative complement pathway, inhibits its conversion to the active molecule C5a, thereby halting pathway progression and membrane attack complex formation [10]. In this case, anti-CFH antibody testing was negative, and ADAMTS13 activity assessment was unavailable. Genetic analysis was recommended but was hindered by financial constraints. Consequently, plasmapheresis was initiated, although eculizumab would have been a preferable choice if accessible.

Severe stage two hypertension in our patient, progressing to encephalopathy, further emphasizes the severity of HUS activity. A 2019 study by Cavero et al. [11] concluded that severe stage two hypertension is common among atypical HUS patients, and eculizumab treatment induces higher renal remission and greater survival compared to plasmapheresis. While our patient responded to plasmapheresis, the role of injection rituximab and immunosuppression remains uncertain, especially with the negative anti-CFH antibody result.

The 2009 European guidelines, predating the era of eculizumab, recommended prompt atypical HUS therapy with plasmapheresis within 24 hours of presentation [12]. However, plasmapheresis was delayed in this case due to an initially confusing clinical scenario, potentially contributing to the delayed response. Since the anti-CFH antibody was negative in our case, the role of rituximab and immunosuppression in inducing remission remains obscured.

Limitations in this case primarily stem from the resource-limited setting, where certain investigations, including ADAMTS13 activity, serum homocysteine levels, and genetic panels, were unavailable. These would have provided crucial etiological clarity and additional treatment guidance. The use of eculizumab could have induced early remission.

## Conclusions

This case highlights the successful management of severe HUS using plasmapheresis. Early initiation of plasmapheresis within 24 hours is crucial for a desired response in severe HUS cases. Confirmatory investigations to rule out secondary causes and a genetic panel are essential before diagnosing atypical HUS. A heightened suspicion of genetic causes is necessary in young children, especially those presenting before and around two years of age. In resource-limited settings, the lack of genetic analysis facilities should not hinder the timely initiation of plasmapheresis treatment.
